# The early minutes of in-hospital cardiac arrest: Shock or CPR? A population based prospective study

**DOI:** 10.1186/1757-7241-16-11

**Published:** 2008-09-22

**Authors:** Eirik Skogvoll, Trond Nordseth

**Affiliations:** 1 Department of Anaesthesiology and Emergency Medicine, St. Olav University Hospital, Olav Kyrres gate 17, N-7006 Trondheim, Norway; 2 Institute of Circulation and Medical Imaging, Faculty of Medicine, Norwegian University of Science and Technology (NTNU), Trondheim, Norway

## Abstract

**Objectives:**

In the early minutes of cardiac arrest, timing of defibrillation and cardiopulmonary resuscitation during the basic life support phase (BLS CPR) is debated. Aims of this study were to provide in-hospital incidence and outcome data, and to investigate the relation between outcome and time from collapse to defibrillation, time to BLS CPR, and CPR quality.

**Methods:**

Resuscitation attempts during a 3-year period at St. Olav's University Hospital (960 beds) were prospectively registered. The times between collapse and initiation of BLS CPR, and defibrillation were determined. CPR quality was assessed by the resuscitation team. The relation between these variables and outcome (short term survival and discharge) was explored using non-parametric correlation and logistic regression.

**Results:**

CPR was started in a total of 223 arrests, an incidence of 77 episodes per 1000 beds per year. Return of spontaneous circulation occurred in 40%, and 29 patients (13%) survived to discharge. Median time from collapse to BLS CPR was 1 minute; CPR was judged to be of good quality in half of the episodes. CPR during the first 3 minutes in ventricular fibrillation (VF/VT) was negatively associated with survival, but later proved beneficial. For patients with non-shockable rhythms, we found no association between outcome and time to BLS or CPR quality.

**Conclusion:**

Our findings indicate that defibrillation should have priority during the first 3 minutes of VF/VT. Later, patients benefit from CPR in conjunction with defibrillation. Patients presenting with non-shockable rhythms have a grave prognosis, and the outcome was not associated with time to BLS or CPR quality.

## Introduction

After in-hospital cardiac arrest, survival to discharge is about 15–20% [1,2]. Key factors determining outcome include the presenting rhythm, time to definite therapy, the episode being witnessed, and provision of basic life support (BLS); understood here as simple airway management, ventilations and external chest compressions i.e. cardiopulmonary resuscitation (BLS phase CPR). The presenting rhythm and time to definite therapy are by far the more important [1-6]. Age, gender, location of arrest, and premorbidity has inconsistently been found to influence survival [3,4].

Hospitals host a high-risk population with better opportunities for data collection and analysis than for out-of-hospital cardiac arrest [7], and time intervals from collapse to BLS and defibrillation are in the order of 1–3 minutes [2,3,5,8]. An earlier study of ours documented an in-hospital incidence of attempted CPR of 59.1 per 1000 beds per year with 17% survival to discharge [9]. The present study was conceived to provide follow-up data, and the advantage of prospective data collection prompted us to explore the relation between outcome and BLS CPR. Ideally, a randomized design would be preferred. On ethical grounds, however, it is difficult to imagine a clinical trial in which CPR quality and defibrillation is intentionally controlled and delayed. When randomized trials are unfeasible, properly planned observational studies addressing patient-important outcomes constitute the next level of evidence [10,11]. Hospital patients reside at different locations, so there is natural variation with respect to BLS CPR as well as time to defibrillation; a situation analogous to the variation in bystander CPR performance observed by paramedics in out-of-hospital cardiac arrest [12].

In-hospital advanced life support (ALS) performance by the cardiac arrest team has recently been considered in detail [13], but BLS CPR performance during the early minutes of in-hospital cardiac arrest has received less attention. Since advanced defibrillators are not attached during the BLS phase, BLS CPR quality cannot be assessed this way.

The 2005 International Consensus Conference on Cardiopulmonary Resuscitation recommended single shocks with maximum energy and interposed CPR rather than serial shocks with escalating energy if ventricular fibrillation (VF) or pulseless ventricular tachycardia (VT) is not terminated immediately [14]. The ideal timing of defibrillation has been debated. Pre-shock CPR has been shown to increase the success of defibrillation after prolonged VF/VT [15-18]. Controversy remains about when rescuers should defibrillate first, or provide CPR first in VF/VT. There was insufficient data to conclude for in-hospital cardiac arrests [19], and the CPR/shock issue has recently been identified as a clinical research priority [20].

The aims of this prospective, population-based observational study was to estimate the incidence and outcome from in-hospital cardiac arrest, and investigate the relation between outcome and time to defibrillation, time to BLS, and CPR quality.

## Methods

### Clinical setting

St. Olav's University Hospital with 960 beds (> 90% occupancy rate) is a tertiary hospital in central Norway, serving a total population of 630 000 with an annual admission rate of about 42 000 patients. In-hospital medical emergencies including cardiac arrests are managed by a resuscitation team consisting of an anaesthesiologist, a medical resident and a nurse anaesthetist. The resuscitation team brings a manual defibrillator (monophasic during the period of study) and adjuncts for ALS. BLS CPR is usually provided by the staff on the wards but defibrillation in VF/VT is rarely done before team arrival, except in the Coronary Care Unit (CCU), Emergency Department (ED) and Intensive Care Unit (ICU). The BLS phase may thus include repeated defibrillation attempts until the resuscitation team has taken over completely.

BLS CPR training for ward personnel is mainly run by nurse anaesthetists who otherwise staff the resuscitation team. During the period of study, BLS and ALS were taught according to the European Resuscitation Council guidelines of 1992 [21,22]. The major differences from today's guidelines were a recommended compression-to-ventilation ratio of 15:2 for BLS, 5:1 for ALS, and multiple defibrillation attempts in VF/VT. As of 1995, ward personnel were taught a ratio of 15:2 at a rate of 100 per minute (instructor E. Bronnes, personal communication).

### Data acquisition and processing

All resuscitation attempts in adults and children involving the resuscitation team in confirmed cardiac arrests (unresponsive, pulseless patients with apnoea or agonal respiration) during the 3-year period from 1st of September 1995 to 31st of August 1998 were included. Every alarm call to the hospital emergency dispatch centre were prospectively registered in a computerized alarm time registry (thus providing a time reference), and tracked by the first author. False alarms, resuscitation at birth, and patients not considered for resuscitation were excluded. The times of patient collapse, start of BLS, resuscitation team arrival, defibrillation, and other relevant resuscitation efforts were estimated to the nearest minute (sometimes second) by the nurse anaesthetist, and registered on a specialised registry form supplementing the anaesthesia record. A digital clock, checked and set weekly, attached to the emergency trolley aided in the registration. If VF/VT was witnessed, and defibrillation performed immediately by ward personnel or the resuscitation team, the collapse-to-defibrillation time interval was set to 0 or 1 minute based on other available information. To avoid spurious accuracy, time intervals were rounded to the nearest minute before analysis.

Upon resuscitation team arrival, BLS CPR performance was assessed with respect to type of CPR (none, ventilation, compressions, or both) and quality (poor or good; as judged by the team from observation of chest inflation and compression depth and rate), and registered on the case sheet as categorical variables. The authors classified CPR quality from the original observations on a 3-point ordinal scale: 0 – No CPR, 1 – Intermediate (i.e. compressions only, ventilations only, or both poorly performed), and 2 – Good quality compressions and ventilations. In nine patients, data were inconsistent and CPR quality was set to 1 (intermediate) by the authors based on other available information. In a few instances when patients had not received BLS CPR, resuscitation was initiated by the resuscitation team on arrival; these were classified as "No BLS CPR". Alternatively, BLS CPR was treated as present (level 2 above) or not (levels 0 and 1 above combined). The Utstein time intervals "collapse-to-first CPR" (T_CPR_) and "collapse-to-first defibrillation" (T_defib_) were the primary variables for analysis [23].

If the recordings from the resuscitation team were incomplete or ambiguous, personnel involved were interviewed by the first author as soon as possible after the episode (usually the same or next day, on weekends usually the next working day) for completion. In particular, ward personnel were interviewed with respect to the time course. Outcome and supplementary data was retrieved from the patient's medical chart, as needed.

For the primary correlation analyses, we employed a five-point ordinal outcome measure [24]: 0 – No response at all; 1 – Signs of life during resuscitation (respiratory gasps, short-lived pulse) but dead on scene; 2 – Return of spontaneous circulation (ROSC) but dead within 24 h; 3 – ROSC > 24 hours but dead before discharge; 4 – Discharge from hospital. For the purpose of visualisation and statistical modelling, the scale was simplified to 1 – No ROSC; 2 – ROSC but not discharged; 3 – Survival (discharged from hospital).

The Regional Committee for Medical Research Ethics was consulted, and decided that formal approval was not required; as the study was observational and involved no experimental intervention. Due to a lack of manpower and financial resources, final analysis of the study was not completed until 2007.

### Statistical methods

The results are reported as mean or median values with standard deviation (SD), interquartile range (IQR), range, or 95% confidence intervals, according to type of variable and approach to the problem. Confidence intervals for binomial parameters were calculated according to Wilson [25]. The relation between the 5-point outcome, time, and CPR quality was explored using exact non-parametric correlation analysis with allowance for ties. Further statistical modelling was done as follows and separately for patients with initial rhythm VF/VT and non-shockable rhythms (asystole or pulseless electrical activity, PEA). Log transformed time was found to give a significantly better model fit than linear time [26]; in effect this transformation sets focus on the lower end of the time scale. Considered to reflect an underlying continuum, CPR quality was entered both as a scale variable with 3 levels (0, 1, or 2) and as a binary variable (0 or 1). Outcome was modelled as a binary variable (0 or 1) in the logistic regression model.

No formal sample size calculation was done; but the observations from 1990 through 1994 [9] suggested about 200 episodes and 30 survivors to be expected during the project's time frame of 3 years. For logistic regression, a ratio between covariates and observations of 1:10 is considered acceptable [27]. Descriptive analysis was done with the SPSS^® ^version 14 (SPSS Inc. Chicago, Ill.), exact correlation analysis with StatExact 8 (Cytel Corp. Cambridge, Ma.), and statistical modelling with the software R, version 2.6.1 [28]. P values less than 0.05 was considered to indicate statistical significance.

## Results

A total of 223 episodes of cardiac arrest occurred in 219 patients, yielding an incidence of 77 per 1000 beds per year. During the period, approximately 2860 patients died and 882 200 patients-days were spent at the hospital, indicating that CPR was instituted in 8% of in-hospital deaths; at a rate of 1.76 per 1000 admissions, or 0.25 episodes per 1000 patient-days. Two episodes were excluded from further analysis as being very atypical and not providing useful information: One patient died from VF in the ED when the defibrillator repeatedly malfunctioned. Another patient arrested in the ED from hypothermia, received cardiopulmonary bypass, and survived.

Among the remaining 217 patients and 221 episodes, the median age was 75 years, 66% were male, and three patients were < 18 years. Cardiac aetiology, i.e. no other obvious cause, was presumed present in 179 patients (81%). The outcome was determined in all patients (table 1). One patient arrested four times and was discharged twice two months apart. Another patient arrested twice, survived for 24 hours but died before hospital discharge. Only the first episode per hospital admission was included in the models to avoid statistical dependency problems.

**Table 1:** Episode and time characteristics vs. outcome

	All episodes* n = 221	Dead on scene n = 132	ROSC n = 57	Survived the episode* n = 32
**Episode characteristics**				
				
**Location of arrest**				
ICU/CCU	27 (12%)	16	8	3 (11%, 4 to 28%)
Other (mainly wards)	160 (72%)	102	40	18 (11%, 7 to 17%)
Emergency department	34 (15%)	14	9	11 (32%, 19 to 49%)
				
**Witnessed**	163 (74%)	94	43	26 (16%, 11 to 22%)
				
**CPR quality**				
2 – Good	123 (56%)	74	35	14 (11%, 7 to 18%)
1 – Intermediate	63 (28%)	41	13	9 (14%, 8 to 25%)
0 – None	35 (16%)	17	9	9 (26%, 14 to 42%)
				
**Presenting rhythm**				
VF/VT	90 (41%)	29	31	30 (33%, 24 to 44%)
Asystole	66 (30%)	49	16	1 (2%, 0 to 8 %)
PEA	65 (29%)	54	10	1 (2%, 0 to 8 %)
				
**Time characteristics**				
				
**Collapse-to-defibrillation, VF/VT**				
Median with IQR (minutes)	4.0 (1.25, 6.75)	6.0 (3.5, 8)	4.0 (2.0, 6.0)	2.0 (1.0, 4.0)
				
**Collapse-to-BLS, VF/VT**				
Median with IQR (minutes)	1.0 (0.0, 2.0)	1.0 (0.0, 2.0)	1.0 (0.0, 1.0)	1.0 (0.0, 2.0)
				
**Collapse-to-BLS, PEA/ASY**				
Median with IQR (minutes)	1.0 (0.0, 2.0)	1.0 (0.0, 2.0)	1.0 (0.0, 4.0)	0.5 (0.25, 0.75)

*The number of episodes (n = 221) is higher than the number of patients (n = 217), as two patients arrested more than once (see text). ** Numbers in parenthesis are percentages with 95% confidence intervals

**Figure 1:**
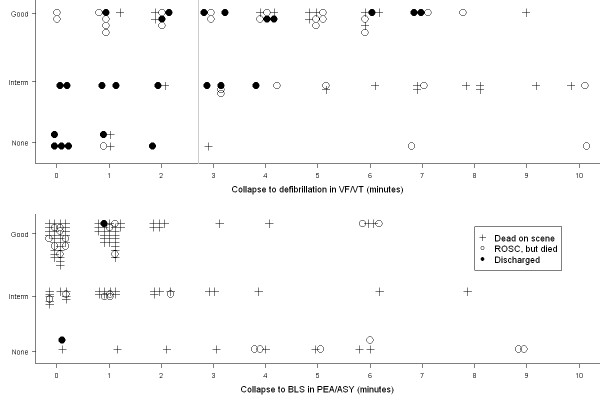
**Relation between outcome, CPR quality and time to first defibrillation (≤ 10 minutes), in patients presenting with VF/VT (a, upper); or asystole/PEA (b, lower).** Note that the BLS phase may extend beyond the first defibrillation. CPR quality scale: None; Intermediate quality; Compressions and ventilations of good quality. Individual observations have been scattered and stacked to improve visualization.

In almost half of the patients there was no response, whereas 12% showed signs of life during resuscitation but eventually died on scene. ROSC was achieved in 40% of the patients, half of whom died within 24 hours. Among the 217 patients, 29 survived to hospital discharge (13%, 95% CI: 9 to 19%); two of whom had presented with asystole or PEA. One-year survival was 9.7% and five-year survival 7.8%. CPR quality was found to be good in about half of the episodes (table 1). Figure 1 presents the observed relation between outcome, time, and CPR quality.

### Presenting rhythm VF/VT

**Figure 2:**
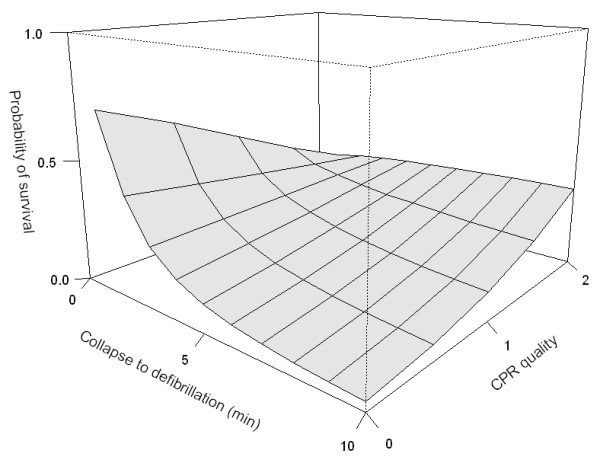
**Estimated probability surface of survival among patients with VF/VT, according to time to first defibrillation (≤ 10 minutes), and BLS CPR quality.** CPR quality scale: 0 – No CPR; 1 – Intermediate quality; 2 – Compression and ventilations of good quality. Logistic regression coefficients with 95% confidence intervals in parentheses: Model r-square = 0.22, Intercept = 0.96 (-0.32 to 2.57), CPR quality = -1.01(-2.09 to -0.10), Log time (min) = -1.87 (-3.37 to -0.72), Interaction term = 1.01 (0.24 to 1.91).

**Figure 3:**
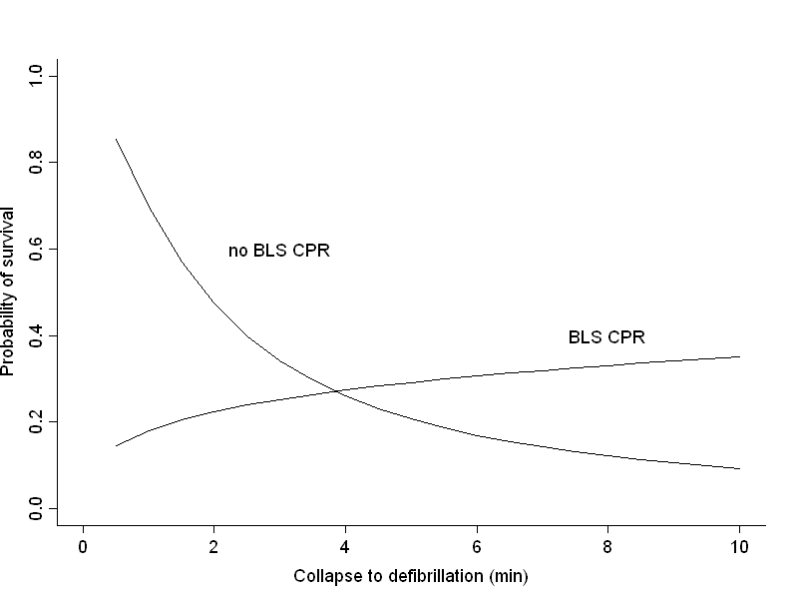
**Estimated probability of survival among patients with VF/VT, according to time to first defibrillation (≤ 10 minutes), and to whether BLS CPR was provided or not.** Logistic regression coefficients with 95% confidence intervals in parentheses: Model r-square = 0.26, Intercept = 0.84 (-0.17 to 2.06), CPR = -2.35 (-4.25 to -0.74), Log (time [min]) = -1.35 (-2.32 to -0.61), Interaction term = 1.74 (0.61 to 3.05).

Median T_defib _was 4 minutes (IQR: 82 – 412 s). We found a negative correlation between outcome and T_defib _(Spearman's rho = -0.38, 95% CI: -0.58 to -0.18, p < 0.001), but not with T_CPR _(Spearman's rho = -0.0127, 95% CI: -0.23 to 0.21, p = 0.90), or with CPR quality (Somer's d = -0.02, 95% CI: -0.18 to 0.15, p = 0.85). In the statistical models, the variables log (T_defib_), CPR quality, and their interaction (i.e. product term) were found to be statistically significant (coefficients given in Figures 2 and 3). This phenomenon is visualized in figure 1a: with T_defib _less than about 3 minutes (figure 1, grey line), survival is better among those who did not receive BLS CPR. When T_defib _exceeds this value, all patients with ROSC appear in the upper two strata of figure 1a, corresponding to CPR of increasing quality. The time point (T_defib_) at which BLS impact changes from negative to positive was calculated to be 2.72 minutes with CPR quality scale 0–2 (Figure 2) or 3.85 min with CPR quality scale 0–1 (Figure 3). Figure 2 shows the response surface derived from the statistical model, with the expected probability of survival according to CPR quality scale 0–2 and time to defibrillation. At T_defib _= 1 minute, the baseline probability of survival is about 70%. If no defibrillator is immediately available and CPR is not provided, survival rapidly decreases to about 3% at T_defib _= 10 minutes. Providing CPR in conjunction with defibrillation at this time increases the probability of survival to about 33%. Immediate CPR at T_defib _= 1 in conjunction with defibrillation is associated with a drop in survival to approximately 25%. The interaction between time and CPR, i.e. how CPR impact changes from negative to positive, can be seen as a twist of the surface. Figure 3 illustrates the same phenomenon when CPR is treated as a binary variable (0–1); the curves intersect close to 4 min.

### Presenting rhythm PEA or ASY

Among the 131 patients with presenting rhythms of asystole and PEA (Figure 1b), 19 (15%) had not received BLS but were resuscitated by the resuscitation team; among these were eight episodes witnessed by the team. Seven of the 19 achieved ROSC and one was discharged. We found no relation between outcome and T_CPR _(Spearman's rho = -0.002, 95% CI: -0.18 to 0.17, p = 0.98), or with CPR quality (Somer's d = -0.04, 95% CI: -0.21 to 0.12, p = 0.58).

A total of 43 patients with a presenting rhythm of asystole or PEA received DC shocks. Among these, 19 had converted to a shockable rhythm during resuscitation and were properly defibrillated; one of them survived to discharge. These episodes were retained and analysed in the PEA/ASY group.

## Discussion

There are two main findings in this population-based study of in-hospital cardiac arrest. First, our findings indicate that defibrillation should have priority during the first 3 to 4 minutes of VF/VT. After this period CPR in conjunction with defibrillation improves survival; an interdependence between BLS CPR and time that this study has been able to visualize and model. Second, we find that BLS with or without ALS is rather ineffective in PEA or asystole, and that the outcome seems to be independent of BLS CPR quality.

### BLS phase CPR quality vs. time to defibrillation in VF/VT

We found a clear association between early defibrillation of VF/VT and survival to discharge, as others have done [1,3-5]. Weisfeldt et al. postulated a "3-phase model" of cardiac arrest, where after 3 minutes the patient enters a "circulatory phase" where BLS may be of more benefit than defibrillation [15]. Cobb et al. [17] and Wik et al [18] suggested benefit from pre-shock CPR in prolonged VF/VT. We found similar results; patients with VF/VT lasting more than 3–4 minutes benefit from CPR. However, as was also seen in the ALS phase model derived by Wik et al. from their randomised study, and in an observational study of witnessed VF [29], patients defibrillated within the first 3–6 minutes had better survival without CPR. The present study visualizes and models this relation even further back in time towards the arrest. The apparent negative effect of early CPR in VF/VT in our study is perhaps surprising and controversial, but nevertheless notable. It is not fully explained by the delayed defibrillation [6] done by the resuscitation team after BLS was already established, since the effect of time should be similar across the CPR strata. An explicit interaction term was needed to model this relation; otherwise it would have gone undetected – as in the initial correlation analysis. We considered whether it might be due to BLS CPR being administered preferably following (an unsuccessful) defibrillation, i.e. with T_defib _≤ T_CPR _(n = 19), but found no evidence for this. Note that Fig. 1 shows the time to the *first *defibrillation in VF/VT, so a number of patients received BLS CPR beyond this time, before the resuscitation team took over. The possibility of a frank negative effect from (very) early CPR in VF/VT must thus be considered. Unfortunately, the observational design of this study prohibits further clarification at this point. But the current recommendation [30] of *always *interposing 2 minutes of CPR after a non-successful shock in VF/VT seems debatable during the first 2–3 minutes of cardiac arrest. Repeated shocks with escalating energy [31] can be an alternative.

### BLS phase CPR quality in PEA and asystole

With presenting rhythms of asystole and PEA, BLS was rather ineffective, and more than 70% never achieved ROSC. Slightly better outcomes have been noted earlier [9] and in other studies [32,33]. When interpreting this dismal result, it is important to note that we only included patients in definite cardiac arrest whose rhythm was verified, i.e. neither syncope nor isolated respiratory arrests. The absence of any association (i.e. zero correlation) between outcome and time to BLS, or BLS CPR quality, is notable. The confidence intervals extend somewhat in both directions from zero due to the limited sample size. Our clinical interpretation is that other factors, likely related to the underlying aetiology, are more important for the outcome than BLS CPR.

### Limitations of the study

The major limitation of this study is the observational design, in which the circumstances provide variation of CPR quality and response times that were investigated with respect to outcome. In principle, causality cannot be inferred, as there may be underlying confounding factors that are also related to the outcome.

Every resuscitation team mission was tracked and the outcome determined in all cases, but we are aware that the resuscitation team was not alerted in some brief, successful defibrillations from VF/VT in the CCU and probably also in the catheterisation lab. If included, these events would most likely have raised the overall survival rate. In the ICU, the resuscitation team was usually summoned in unexpected arrests; but a small number of resuscitation attempts may have escaped registration. We acknowledge some subjectivity when determining the time course, in particular with respect to the time of collapse. A small number of conflicting observations had to be reconciled by the investigators prior to analysis. However, the absolute time inaccuracies are likely to be small within the narrow time frame explored, and in most episodes the *order *of events could be determined with reasonable certainty. Rather than focusing on "numbers", we emphasize visualization.

Assessment of BLS CPR quality is admittedly subjective, but was carried out by experienced and skilled BLS instructors. This was seen as the only realistic option. Even an independent observer would not always be the first on scene; video surveillance is unavailable, and monitoring equipment – even if in use – would not capture BLS phase of CPR quality. Furthermore, the fundamental relation between CPR quality and outcome proved similar, whether a scaled or more robust binary CPR quality measure was employed (Figures 2 and 3).

One may finally question the relevance and validity of observations approaching 10 years of age. Clinical research in this field is time-consuming, and treatment recommendations rely on accumulating clinical evidence over years as well as extrapolation from animal- and simulation studies. The present study refers directly to the population it concerns; although comorbidity among today's hospital patients may have increased as more patients are now treated on an out-patient basis. The fundamental issues of defibrillation timing and CPR quality are currently topics of great interest. In fact, the delay between data acquisition and final analysis enabled an explicit consideration of this interdependence, as highlighted by intervening research. Virtually every combination of CPR quality and time to defibrillation (or time to BLS for the PEA/ASY group) during the early minutes was observed in our study, allowing for efficient model estimation. One may still wonder whether different results would have emerged following implementation of the more recent CPR guidelines. We find this unlikely for a number of reasons. First, whether defibrillation or BLS comes first is determined by circumstances rather than regulations, and in this turmoil strict protocols are rarely adhered to. Second, there will always be variation with respect to BLS performance; uniform excellence is unrealistic. Third, in contrast to ALS guidelines the BLS guidelines changed only recently (in 2005), and the increase in compression rate from 80–100 to 100 per minute recommended in 1998 [34] was already implemented. Finally, the clinical presentation and outcome from in-hospital resuscitation at our institution remains essentially unaltered [35].

## Conclusion

Our findings indicate that defibrillation should have priority during the first 2–3 minutes of VF/VT. After this time, patients benefit from CPR in conjunction with defibrillation. Patients presenting with PEA or asystole have a grave prognosis, and the outcome was not associated with time to BLS, or BLS CPR quality.

## Competing interests

The authors declare that they have no competing interests.

## Authors' contributions

ES designed the study and performed data collection, had access to all data, performed statistical modelling and drafted the manuscript. TN carried out final outcome analysis, cross-checked data quality and participated at all stages during manuscript preparation. All authors read and approved the final manuscript.

## Funding sources

The study was funded by a University research scholarship.
